# Enhancing Deep Learning Forecasts with Wavelet Decomposition: Evidence from the Ghana Stock Exchange

**DOI:** 10.3390/e28070782

**Published:** 2026-07-09

**Authors:** Osei K. Tweneboah, Maria C. Mariani

**Affiliations:** 1 Ramapo Data Science Program, Ramapo College of New Jersey, Mahwah, NJ 07430, USA; otwenebo@ramapo.edu; 2 Department of Mathematical Sciences, The University of Texas at El Paso, El Paso, TX 79968, USA

**Keywords:** emerging markets, Ghana Stock Exchange Composite Index, volatility, deep learning models, wavelet transformation, stock market prediction

## Abstract

Forecasting stock market returns in emerging economies remains challenging due to market volatility, structural irregularities, and limited data availability. This study investigates whether discrete wavelet transformation can enhance the predictive performance of deep learning models when applied to financial time series from emerging markets. Using daily returns of the Ghana Stock Exchange Composite Index (GSE-CI) spanning 2011 to 2022, we evaluate three widely used deep learning architectures—Multilayer Perceptron (MLP), Long Short-Term Memory (LSTM), and Convolutional Neural Network (CNN)—in both their standard form and with preprocessing based on the Daubechies-4 (db4) discrete wavelet transform. The empirical results indicate that wavelet preprocessing consistently reduced forecasting errors across all three deep learning architectures, highlighting its effectiveness as a multiscale feature extraction and noise reduction technique for financial time series. Among the models considered, the Wavelet-LSTM achieved the lowest forecasting error, while the wavelet-enhanced variants consistently outperformed their corresponding baseline models. These findings suggest that the benefits of wavelet decomposition extend beyond a specific neural network architecture by providing a richer representation of nonlinear temporal dynamics in volatile and data-constrained financial environments. As one of the first studies to systematically evaluate wavelet-augmented deep learning models for stock market forecasting in an African equity market, this work contributes to the growing literature on hybrid forecasting frameworks and provides practical insights for researchers, analysts, and investors interested in forecasting emerging financial markets.

## 1. Introduction

Emerging financial markets offer substantial growth opportunities but are often characterized by high volatility, market inefficiencies, limited liquidity, and structural instability. These characteristics make forecasting particularly challenging, as price dynamics are frequently influenced by abrupt policy changes, information asymmetries, and external economic shocks. Unlike developed markets, where extensive historical data and relatively stable market structures facilitate modeling efforts, emerging markets often exhibit nonlinear and nonstationary behavior that can reduce the effectiveness of conventional forecasting approaches. As noted by Harvey [1], such market inefficiencies may create opportunities for predictive modeling, provided that forecasting techniques are capable of capturing the unique dynamics inherent in these environments.

Traditional econometric approaches have long been employed to model financial market behavior. Models such as ARCH and GARCH have proven useful for characterizing volatility clustering and conditional heteroskedasticity [2,3,4]. However, these methods rely on restrictive assumptions and are often less effective in capturing complex nonlinear relationships, structural breaks, and evolving temporal dependencies that are frequently observed in emerging markets. Consequently, there has been growing interest in more flexible, data-driven approaches capable of learning complex patterns directly from financial time series data.

Recent advances in machine learning and deep learning have provided new opportunities for financial forecasting. Architectures such as Multilayer Perceptrons (MLPs), Long Short-Term Memory (LSTM) networks, and Convolutional Neural Networks (CNN) have demonstrated promising performance in modeling nonlinear and sequential relationships in financial datasets [5,6]. Nevertheless, the performance of these models often depends heavily on the quality of the input data. Financial time series are typically noisy and contain information operating at multiple temporal scales, making feature extraction and signal representation critical components of the forecasting process.

One approach that has gained attention in recent years is the use of wavelet transformation as a preprocessing technique. Wavelet decomposition separates a time series into components representing different frequency bands, enabling the extraction of both short-term fluctuations and long-term trends while simultaneously reducing noise. By transforming the original series into a multiscale representation, wavelet methods can provide a richer set of features for machine learning algorithms to learn from. Previous studies have demonstrated the usefulness of wavelet preprocessing in financial forecasting applications [7]; however, evidence regarding its effectiveness across multiple deep learning architectures remains limited, particularly within the context of emerging financial markets.

From a theoretical perspective, wavelet decomposition offers a multiresolution view of financial time series by capturing information in both the time and frequency domains at once. Unlike Fourier-based methods, which assume data is stationary and use global basis functions, wavelets use localized basis functions that can detect sudden changes, transient events, and nonlinear market movements. This characteristic is especially useful for financial returns, where volatility clustering, structural breaks, and multiscale behaviors are common. By splitting the original series into approximation (low-frequency) and detail (high-frequency) parts, wavelet preprocessing reduces noise while keeping important temporal features, leading to more useful inputs for deep learning models.

In a recent study, Tweneboah et al. [8] investigated the Ghana Stock Exchange Composite Index (GSE-CI) using fractal analysis and Bayesian stochastic volatility models, demonstrating the presence of complex volatility dynamics and long-memory characteristics within the market. While that work focused primarily on volatility characterization and modeling, the present study shifts attention toward forecasting and predictive performance.

The primary objective of this research is to examine whether wavelet decomposition can enhance the forecasting performance of deep learning models in an emerging market environment. Using daily returns from the Ghana Stock Exchange Composite Index (GSE-CI) spanning the period 2011–2022, we evaluate three widely used deep learning architectures—Multilayer Perceptron (MLP), Long Short-Term Memory (LSTM), and Convolutional Neural Network (CNN)—both in their standard form and in combination with discrete wavelet transformation. Rather than focusing solely on identifying the best-performing neural network, this study investigates whether wavelet preprocessing consistently improves predictive accuracy across different model architectures.

### 1.1. Related Work and Contribution

Although deep learning methods have been widely studied in developed financial markets, relatively few studies have examined their application within African stock markets. Existing research on wavelet-based forecasting has generally focused on individual model architectures or developed-market datasets, leaving a gap in understanding how wavelet decomposition influences the performance of different deep learning frameworks in emerging economies.

This study contributes to the literature in three important ways. First, it provides one of the few comprehensive evaluations of wavelet-enhanced deep learning models using data from an African equity market. Second, it systematically compares the effect of wavelet preprocessing across three fundamentally different neural network architectures: MLP, LSTM, and CNN. Third, it demonstrates that wavelet decomposition serves as an effective feature extraction and noise reduction mechanism, leading to consistent improvements in forecasting performance across all architectures considered.

By focusing on the interaction between signal-processing techniques and deep learning models, this research provides new insights into the development of hybrid forecasting frameworks for volatile and data-constrained financial environments.

### 1.2. Paper Structure

The remainder of this paper is organized as follows. Section 2 describes the dataset and preliminary exploratory analysis. Section 3 presents the deep learning architectures and wavelet decomposition methodology. Section 4 reports the empirical forecasting results and comparative performance analysis. Section 5 discusses the implications of the findings for financial forecasting in emerging markets. Finally, Section 6 concludes the study and outlines directions for future research.

## 2. Materials

In this section, we provide a concise overview of the dataset utilized for the modeling and analysis purposes.

### 2.1. Data Collection and Analysis

The data used for this research was sourced from the Ghanaian Stock Exchange Composite Index (GSE-CI), functioning as a key benchmark for stock market performance. This index evaluates performance by considering the combined market capitalization of all listed stocks. Our analysis encompassed daily closing index values collected over a span of twelve years, ranging from 2011 to 2022.

In our study, we opted to work with the natural logarithm of daily returns derived from the GSE-CI closing index. This choice was influenced by the mathematical convenience highlighted by [9]. Logarithmic returns possess the advantage of simple summation over a given number of periods, providing the total return across those periods. Furthermore, when dealing with near-zero simple returns, logarithmic returns offer a close approximation.

(1) $$\begin{array}{cc} \; & R_{t}=\ln(\frac{I_{t}}{I_{t-1}}) \end{array}$$

where $R_{t}$ denotes the return at time t, $I_{t}$ represents the Composite Index at time t, and $I_{t-1}$ signifies the Composite Index at time t − 1.

### 2.2. Data Inspection and Visualization

In the prior research conducted by the authors, which utilized the same dataset and time frame, they observed specific descriptive statistics related to the daily returns of the GSE-CI. The descriptive statistics characterized the daily returns of the GSE-CI, showcased an average return of 0.03% per day during the 12-year period from 2011 to 2022. The high standard deviation of 0.00670 indicates substantial volatility within the GSE-CI, aligning with the financial principle that higher volatility often corresponds to higher expected returns. Additionally, a skewness of 0.5 suggests a nearly symmetrical distribution, while the high kurtosis of 11.9 implies heavy tails, indicating the likelihood of more frequent extreme outcomes than expected by a normal distribution. Consequently, this validates the authors’ choice to employ a stochastic volatility model capable of capturing outlier effects in the series.

**Figure 1:**
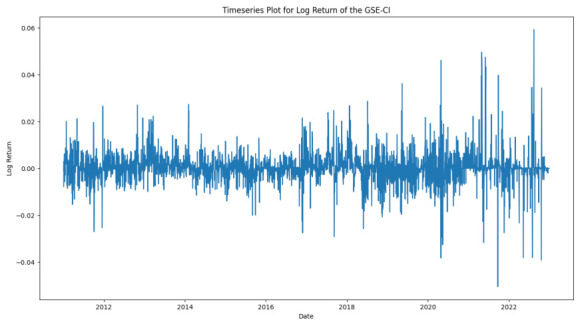
Time Series Plot of the Daily Returns of the GSE-CI.

Figure 1 displays the time series plots for the daily GSE-CI and its corresponding returns. Notably, there is a pronounced surge in volatility observed between 2018 and 2022, contrasting with the earlier period spanning from 2011 to 2017. This heightened volatility aligns with reports from the Ghana Stock Exchange, indicating an uptick in market liquidity during this period. This shift in volatility might be attributed to the government’s decision to restructure the financial sector which caused a lot of panic in the market prompting investors to shift their attention towards money markets.

During the historical analysis, it was found that the GSE-CI achieved its highest recorded return of 6% in August 2022 and its lowest return of −5% in September 2021. Interestingly, the top 5 highest and lowest returns within the study’s timeframe occurred specifically from 2018 to 2022. It’s noteworthy that this period of increased volatility coincides with the period of heightened liquidity. Empirical data sourced from the Ghana Stock Exchange highlights an 18% surge in liquidity in 2019 compared to the preceding year, with 200 million shares traded during that time frame.

**Figure 2:**
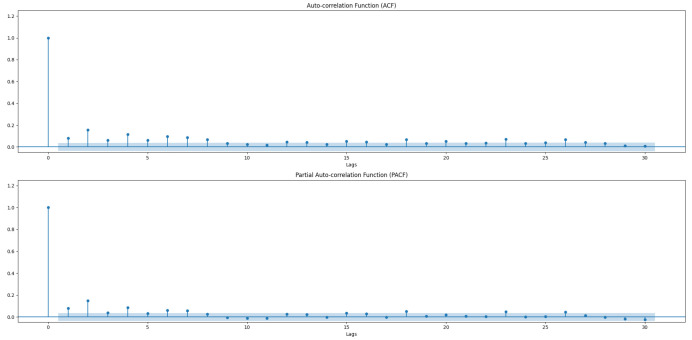
ACF & PACF of the Daily Returns of the GSE-CI.

The ACF measures the correlation between a time series and its lagged values, while the PACF captures the correlation between a time series and its lagged values, excluding the influence of intermediate lags. Analyzing the ACF plot (Figure 2), we observed insignificance beyond lag 7. This implies that, after accounting for the first 7 lags, the subsequent lags do not contribute significantly to the correlation structure of the time series. In other words, the relationship between observations becomes negligible after a lag of 7 time periods. Similarly, the PACF plot showed insignificance beyond lag 6. This signifies that once we account for the first 6 lags in the series, the correlation at longer lags becomes statistically non-significant. These findings suggest a rapid decay in autocorrelation as the lag increases, indicating a short memory in the time series data. Such decay in significance could be attributed to various factors. It might be indicative of a seasonal pattern present in the data, where correlations are strong within certain periodic intervals and diminish outside those intervals. Alternatively, it could signify a lack of long-term memory or persistence in the time series, with observations becoming increasingly independent as time progresses.

**Figure 3:**
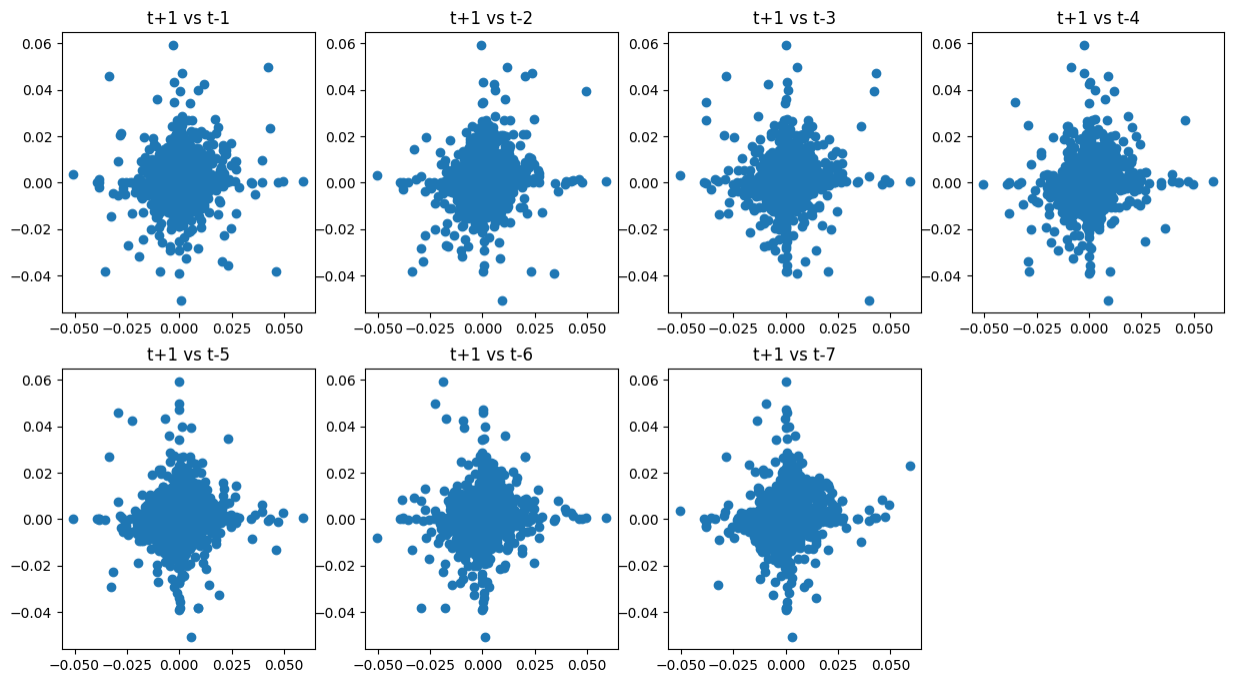
Scatter Plot of Lags of the Daily Returns of the GSE-CI.

The utilization of lagged scatter plots seen in Figure 3 is a powerful technique in time series analysis, providing a visual representation of the relationship between a variable and its lagged values. In our investigation, the scatter plots were constructed for lags up to 7, systematically examining the correlations between lag 1 and lag 2, lag 1 and lag 3, and so forth. Surprisingly, each scatter plot revealed a consistent pattern of positive weak correlations, shedding light on the temporal dynamics embedded in the dataset. This behavior may reflect certain underlying patterns or periodicities in the data, contributing to the observed positive correlations.

## 3. Methods and Modeling

### 3.1. Neural Network

Neural networks, consist of interconnected layers of artificial neurons designed to mimic the workings of the human brain. The journey through a neural network begins with the input layer, where data is received and processed. This information then flows through hidden layers, where complex computations occur, thanks to weighted connections and activation functions that introduce non-linearity. Eventually, the processed data reaches the output layer, where the final predictions or classifications are made. Throughout this journey, the network adjusts its internal parameters, namely the weights and biases, through a process called back-propagation, minimizing the error between predicted and actual outputs. This iterative training process continues until the network learns to make accurate predictions, making it a powerful tool for tasks such as image recognition, natural language processing, and modeling time series data. Utilizing neural networks offers the advantage of resilience to noise within input data and the mapping function, allowing for seamless learning and prediction in the presence of missing values. Moreover, neural networks lack strong assumptions about the mapping function, enabling them to adeptly capture both linear and nonlinear relationships [10].

**Figure 4:**
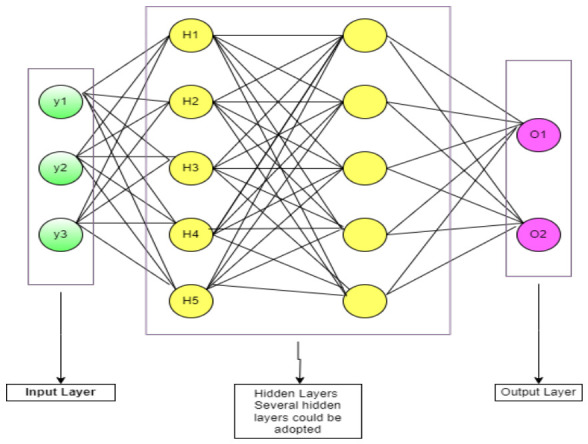
Schematic representation of a multilayer neural network architecture. The green nodes represent the input layer containing the predictor variables, the yellow nodes represent the hidden layers where nonlinear feature learning occurs, and the purple nodes represent the output layer that generates the predicted response. The black lines indicate weighted connections between neurons that are optimized during network training.

1. **Input Layer:** This initial layer serves as the neural network’s entry point, forwarding input values to the subsequent layer without applying any operations. It lacks associated weights and biases. Within this network (see Figure 4), three input signals, denoted as $y_{1},y_{2},y_{3}$, are utilized.
2. **Hidden Layers:** Hidden layers consist of neurons (nodes) that apply diverse transformations to input data. Each hidden layer forms a vertical stack of neurons. In the given image, two hidden layers are depicted: the first with 5 neurons and the second with the same number. The final hidden layer forwards values to the output layer. Importantly, all neurons within a hidden layer are fully interconnected with every neuron in the subsequent layer.
3. **Output Layer:** As the concluding layer in the network, it receives input from the last hidden layer. Through this layer, we can obtain values within a specified range. In our network (see Figure 4), the output layer consists of 2 neurons, producing outputs labeled as o1 and o2.

We employed three distinct neural network architectures for our analysis:

1. Multilayer Perceptron (MLP): A feedforward neural network consisting of an input layer, multiple hidden layers, and an output layer. The MLP was designed with hidden layers, each comprising neurons, and utilized an activation function [11].
2. Long Short-Term Memory (LSTM): A network architecture particularly suited for sequential data, capable of using its internal state (memory) to process variable length sequences of inputs. Our LSTM model was configured with layers and units per layer.
3. Convolutional Neural Network (CNN): Although predominantly used in image recognition tasks, a one-dimensional CNN was adapted for this time-series analysis. The CNN model consisted of convolutional layers with and dense layers. CNNs possess the capability to autonomously learn and extract features from input data, which can be harnessed in the realm of time series prediction. By treating a sequence of objects akin to a one-dimensional image, a CNN model can interpret the most pertinent elements. A distinguishing aspect of CNNs is their utilization of diverse process element, each contributing to an effective representation of local signal importance. Moreover, the complex framework facilitates the stacking of successive strata of these processing units, enabling the deep learning model to characterize importance across varying scales [12].

### 3.2. Model Implementation and Training Configuration

To enhance the reproducibility of the proposed framework, the deep learning models were trained using a standard training configuration whenever applicable. Before model training, the logarithmic return series was normalized with Min–Max scaling. For the wavelet-enhanced models, the normalized series was first transformed using the Discrete Wavelet Transform (DWT), and the resulting wavelet coefficients served as input features. A sliding-window approach with a seven-observation look-back period was used to create the supervised learning sequences for all models.

The networks were optimized using the Adam optimizer with the Mean Squared Error (MSE) loss function. A batch size of 32 and 100 training epochs was used throughout the experiments. The default Adam learning rate of 0.001 was adopted. The LSTM architecture consisted of two stacked LSTM layers containing 128 and 64 memory units, respectively, followed by a dense output layer. The remaining neural network architectures (MLP and CNN) were trained using the same preprocessing pipeline and optimization settings to ensure a consistent comparison of the effect of wavelet preprocessing across different deep learning models.

The LSTM implementation employed the default TensorFlow/Keras activation functions, namely the hyperbolic tangent (tanh) activation for the memory cell and the sigmoid activation for the recurrent gating mechanisms. A single dense output neuron was used to generate one-step-ahead forecasts.

The hyperparameters presented in Table 1 correspond to the LSTM implementation. To ensure a fair evaluation of the impact of wavelet preprocessing, the MLP and CNN models employed the same data preprocessing pipeline, including Min–Max normalization, the same train–test partition, optimization strategy (Adam), loss function (MSE), training epochs, batch size, and look-back window where applicable. The primary differences among the models lie in their internal network architectures, while the experimental protocol remained consistent across all deep learning models.

### 3.3. Discrete Wavelet Transformation

The Discrete Wavelet Transform (DWT) was used as a preprocessing method to improve the representation of the financial time series before model training. Unlike traditional signal processing methods that analyze data solely in the frequency domain, DWT offers simultaneous localization in both time and frequency, making it especially suitable for nonlinear and nonstationary financial data. The transformation breaks down the original return series into multiple frequency bands, enabling separate analysis of long-term market trends and short-term fluctuations. In this study, the Daubechies-4 (db4) wavelet was chosen for its excellent localization properties and its capacity to represent financial signals with sudden changes and multiscale features. A three-level wavelet decomposition was performed using the pywt.wavedec() function, resulting in one approximation coefficient and three sets of detail coefficients. The approximation coefficients capture the low-frequency trend of the series, while the detail coefficients reflect progressively higher-frequency market variations. The approximation and detail coefficients were concatenated to create a multiscale feature representation, normalized through Min–Max scaling, and then used as inputs for the deep learning models.

By harnessing the unique attributes of Daubechies wavelets, analysts can proficiently extract meaningful insights, detect anomalies, and discern temporal trends, thereby enhancing the effectiveness of time series analysis methodologies, See [13,14,15].

The general equation for the Discrete Wavelets Transform is given by:

(2) $$D\left[a,b\right]=\frac{1}{\sqrt{b}}\sum_{m=0}^{p-1}f\left[t_{m}\right]\underset{\mathrm{wavelet}}{\underset{︸}{\psi [\frac{t_{m}-a}{b}]}}$$

where

$a=k2^{-j}$ known as the Approximation Coefficient;
$b=2^{-j}$ known as the Detail Coefficient;
ψ= scale parameter;
k= wavelet transform signal index.

**Figure 5:**
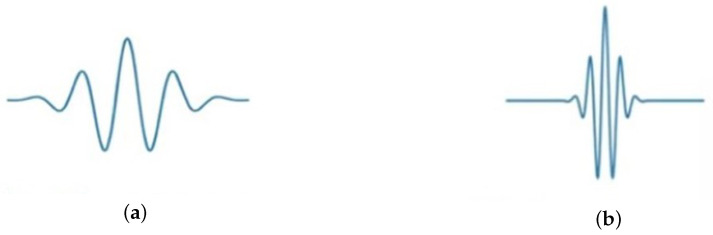
(**a**) Expanded wavelet for resolving low-frequency components. (**b**) Shrunken wavelet for resolving high-frequency components.

When the scale parameter (ψ) of a wavelet expands (see Figure 5a), it essentially widens the window of observation, allowing the wavelet to capture lower frequency components of the signal more effectively. This expanded wavelet is particularly adept at detecting and representing low-frequency variations or trends present in the data. On the other hand, when the scale parameter shrinks (See Figure 5b), the wavelet’s window of observation becomes narrower, enabling it to focus more on high-frequency details within the signal. This shrunken wavelet excels at capturing rapid changes or fine-scale features in the data. In essence, the behavior of the wavelet when the scale parameter expands determines its ability to resolve low-frequency components of the signal, providing valuable insights into the underlying trends or patterns present in the data.

Illustration of wavelet scaling for frequency resolution. An expanded wavelet captures low-frequency components and long-term trends, whereas a shrunken wavelet captures high-frequency components and short-term fluctuations.

### 3.4. Discrete Wavelet Neural Network

**Figure 6:**
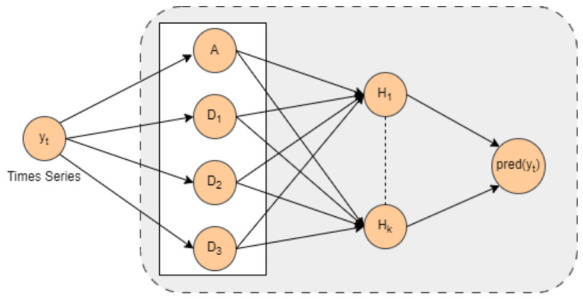
Wavelet Neural Network Architecture.

In this study, the wavelet transforms are utilized to decompose signals into approximation coefficients, which capture short-term variations, and detailed coefficients, which capture long-term trends. These coefficients serve as input layers for the neural network models employed in the analysis, as illustrated Figure 6. By leveraging the distinct capabilities of approximation and detailed coefficients, the neural networks are empowered to effectively learn and extract both short-term and long-term patterns from the data. This approach not only enhances the interpretability of the model but also enables the extraction of comprehensive insights into the underlying dynamics of the signal. By integrating wavelet decomposition with neural network architectures, the study aims to achieve a more nuanced understanding of the complex temporal structures inherent in the data, thereby advancing the capabilities of time series analysis methodologies.

where
$y_{t}=$ the time series data (daily returns on the stock market);
A= known as the Approximation Coefficient;
$D_{n}=$ known as the Detail Coefficient n=1,2,3;
H= hidden layers;
$pred(y_{t})=$ output layer.

### 3.5. Wavelet-LSTM Forecasting Pipeline

The proposed Wavelet-LSTM framework combines multiscale signal decomposition with long short-term memory learning. The preprocessing pipeline consists of the following sequential steps:

1. Compute the logarithmic daily returns of the GSE-CI.
2. Normalize the return series using Min–Max scaling.
3. Apply a three-level Discrete Wavelet Transform (DWT) using the Daubechies-4 (db4) wavelet.
4. Extract the approximation coefficient together with the detail coefficients generated by the decomposition.
5. Concatenate the approximation and detail coefficients to form a multiscale feature representation.
6. Construct supervised learning sequences using a sliding window with a look-back period of seven observations.
7. Train the LSTM network using the transformed feature representation with the Adam optimizer and Mean Squared Error (MSE) loss function.
8. Generate one-step-ahead forecasts of the stock market returns.

The term *Wavelet-LSTM* therefore refers to an LSTM forecasting model whose inputs consist of wavelet-transformed features rather than the original time series. The same preprocessing strategy was applied to the Wavelet-MLP and Wavelet-CNN models to ensure a consistent evaluation of the effect of wavelet decomposition across different deep learning architectures.

## 4. Results

This section presents the forecasting performance of six deep learning models applied to the Ghana Stock Exchange Composite Index (GSE-CI): Multilayer Perceptron (MLP), Long Short-Term Memory (LSTM), and Convolutional Neural Network (CNN), together with their wavelet-enhanced counterparts. The primary objective is to evaluate whether discrete wavelet transformation serves as an effective preprocessing technique for improving forecasting accuracy in an emerging market setting.

The dataset was partitioned into a training set (60%), spanning 4 January 2011 to 3 March 2018 (1783 observations), and a testing set (40%), spanning 4 March 2018 to 30 December 2022 (1190 observations). All models were trained using the training set and evaluated on the testing set.

### 4.1. Model Performance Evaluation

Table 2 presents the Root Mean Square Error (RMSE) values obtained from each forecasting model. A consistent pattern emerges across all three architectures: the incorporation of wavelet decomposition resulted in lower forecasting errors relative to their corresponding baseline models. Specifically, the RMSE of the MLP decreased from 0.008632 to 0.008358, the LSTM improved from 0.008232 to 0.008210, and the CNN improved from 0.008297 to 0.008223.

These results show that wavelet preprocessing consistently reduced forecasting errors across all three deep learning architectures. Although the numerical improvements in RMSE are fairly small, reductions were observed across all models considered, suggesting that the improvement stems from the multiscale feature extraction of wavelet decomposition rather than any specific neural network architecture. In financial forecasting, where improving predictive performance is challenging due to the noisy and unpredictable nature of market data, even small and consistent declines in forecasting error can be meaningful improvements in model performance.

Among all models considered, the Wavelet-LSTM achieved the lowest RMSE value of 0.008210, indicating the strongest overall forecasting performance.

### 4.2. Visual Diagnostics

**Figure 7:**
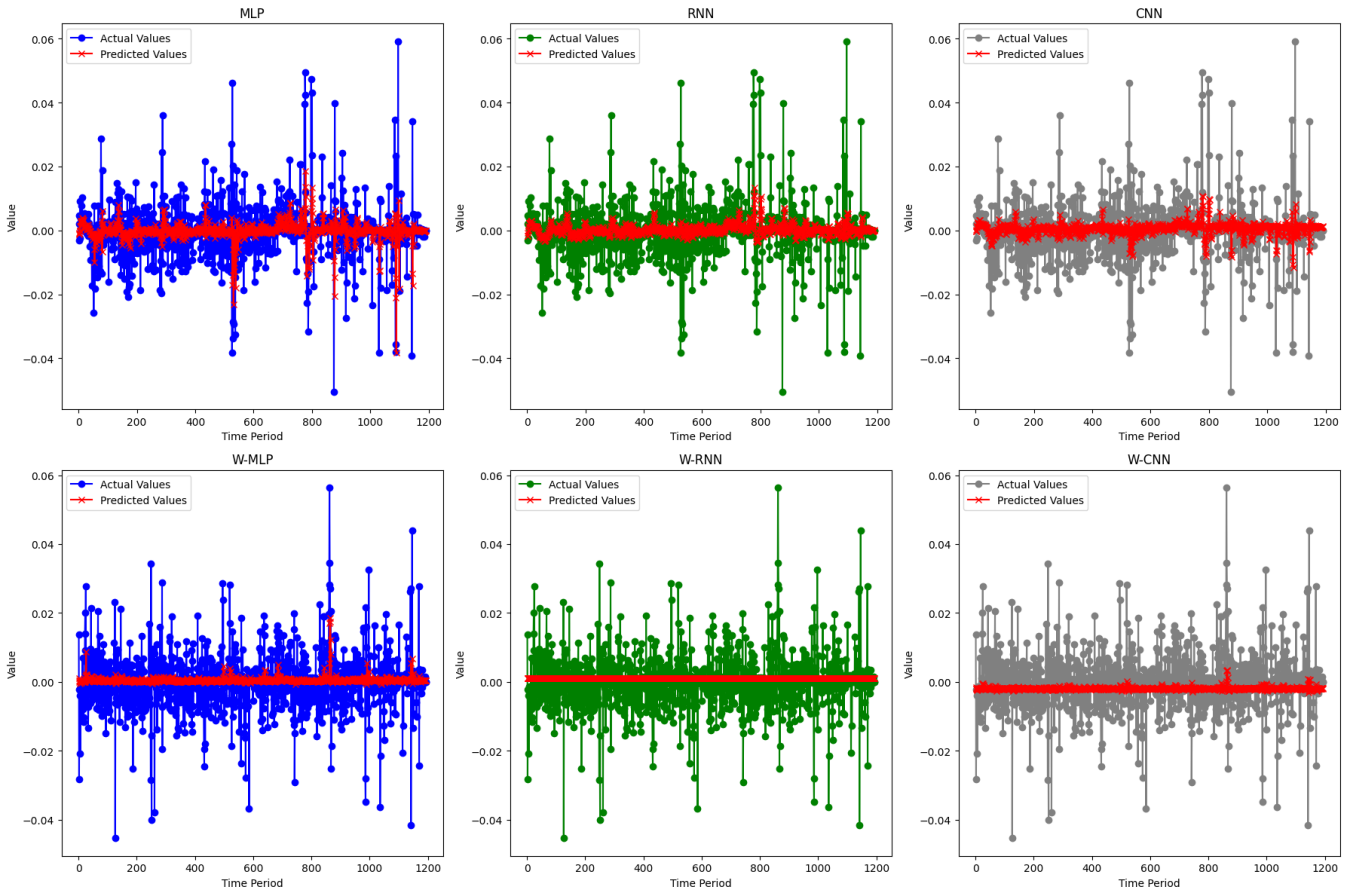
Predicted versus observed values for the six forecasting models.

Figure 7 illustrates the relationship between observed and predicted values for each model. Across all architectures, the wavelet-enhanced versions exhibit tighter clustering around the reference line, indicating improved agreement between actual and predicted returns. The Wavelet-LSTM model displays the most concentrated distribution, consistent with its superior quantitative performance.

The visual evidence complements the numerical results, reinforcing the conclusion that wavelet preprocessing enhances the ability of deep learning models to capture the complex dynamics embedded within the GSE-CI return series.

### 4.3. Key Findings

Three principal findings emerge from the analysis. First, wavelet decomposition consistently improved forecasting accuracy across all deep learning architectures considered in this study. Second, LSTM-based models achieved the strongest overall performance, reflecting their ability to capture temporal dependencies in sequential financial data. Third, the combination of wavelet decomposition and LSTM architecture produced the lowest forecasting error, suggesting that multiscale signal decomposition and memory-based learning provide complementary advantages for stock market prediction.

Taken together, these findings demonstrate that wavelet transformation can serve as an effective feature-engineering and noise-reduction mechanism for deep learning models operating in volatile and data-constrained financial environments.

## 5. Discussion

The primary objective of this study was not merely to identify the best-performing deep learning architecture, but rather to investigate whether wavelet decomposition can systematically improve forecasting performance across different neural network frameworks in an emerging market environment. The results provide consistent evidence in support of this hypothesis.

Although the reductions in forecasting error are relatively small in absolute magnitude, they were consistently observed across all three deep learning architectures. This consistency is an important finding because financial return series are inherently noisy, highly stochastic, and difficult to predict. In such environments, substantial improvements in forecasting accuracy are uncommon, and incremental gains obtained consistently across multiple independent model architectures provide evidence that the preprocessing methodology contributes positively to model performance. Rather than emphasizing the magnitude of improvement achieved by a single model, the present study demonstrates that wavelet decomposition serves as a general feature-extraction mechanism that enhances diverse deep learning frameworks.

One possible explanation for this improvement is that wavelet decomposition separates the original financial series into multiple frequency components, thereby reducing noise and allowing the models to learn patterns at different temporal scales. Financial time series are often characterized by nonlinear behavior, abrupt structural changes, and overlapping short- and long-term dynamics. By decomposing the signal into more interpretable components, wavelet transformation provides a richer representation of the data that can be exploited by deep learning algorithms.

Among the models examined, the Wavelet-LSTM achieved the lowest forecasting error. This result is consistent with the well-established ability of LSTM networks to model temporal dependencies and retain relevant information across time. When combined with wavelet decomposition, the model benefits from both multiscale feature extraction and sequential memory mechanisms, yielding improved predictive performance.

These findings are particularly relevant in the context of emerging markets such as Ghana, where data limitations, lower liquidity, and heightened volatility present significant forecasting challenges. The results indicate that preprocessing techniques may play an important role in enhancing model performance in such environments, potentially yielding greater benefits than would be observed in more mature and information-efficient markets.

From a methodological perspective, this study contributes to the growing literature on hybrid forecasting frameworks by demonstrating that wavelet decomposition can function as a robust and architecture-independent enhancement to deep learning models. For practitioners and researchers, the findings suggest that incorporating wavelet-based feature extraction may provide a practical means of improving forecasting accuracy when working with noisy and nonstationary financial data.

Overall, the study highlights the value of combining signal-processing techniques with modern machine learning approaches and underscores the importance of developing forecasting methodologies tailored to the unique characteristics of emerging financial markets.

## 6. Conclusions and Future Work

This study investigated the effectiveness of discrete wavelet transformation as a preprocessing technique for enhancing deep learning-based stock market forecasting in an emerging market setting. Using daily returns from the Ghana Stock Exchange Composite Index (GSE-CI), we evaluated three widely used deep learning architectures—Multilayer Perceptron (MLP), Long Short-Term Memory (LSTM), and Convolutional Neural Network (CNN)—both in their standard form and in combination with wavelet decomposition.

The empirical results demonstrate that wavelet preprocessing consistently reduced forecasting errors across all three deep learning architectures considered in this study. Although the numerical improvements were modest, the consistent reduction in prediction error across multiple neural network frameworks supports the use of wavelet decomposition as an effective feature-extraction and noise reduction technique for financial time-series forecasting.

Among the models considered, the Wavelet-LSTM achieved the lowest forecasting error, indicating that the combination of multiscale signal decomposition and memory-based learning is particularly effective for modeling financial time series. However, the broader contribution of this study lies in demonstrating that wavelet-enhanced inputs improve predictive accuracy across diverse deep learning frameworks, highlighting the value of signal-processing techniques as a general enhancement strategy for financial forecasting.

These findings are especially relevant for emerging and frontier markets, where data limitations, market inefficiencies, and heightened volatility often reduce the effectiveness of conventional forecasting approaches. The results suggest that hybrid frameworks combining wavelet decomposition with deep learning can provide more robust and adaptive forecasting tools for such environments.

While the findings show that wavelet preprocessing consistently enhances forecasting performance across the evaluated deep learning architectures, several limitations need recognition. First, the empirical analysis relies solely on the Ghana Stock Exchange Composite Index (GSE-CI), so the conclusions should be viewed within the context of this emerging market. Although the GSE-CI offers a suitable case study for testing forecasting methods in frontier markets, future research should examine the proposed framework with additional African and emerging market indices to determine its wider applicability. Second, the study used a single chronological train–test split to maintain the temporal order of the financial time series. While this method reflects a realistic forecasting scenario, alternative validation techniques, such as rolling-window or walk-forward validation, could provide further insights into the robustness and stability of the methodology across different market conditions. Lastly, this research concentrated on assessing the impact of wavelet preprocessing across various deep learning architectures rather than performing an exhaustive comparison with all possible forecasting techniques. Future studies may expand on this framework by exploring other preprocessing methods, different wavelet families and decomposition levels, transformer-based architectures, and broader cross-market validation efforts.

## Figures and Tables

**Table 1:** Training configuration and hyperparameters for the LSTM implementation. The MLP and CNN models were trained using the same preprocessing pipeline, optimization strategy, and general training settings where applicable to ensure a consistent comparison of the effect of wavelet preprocessing.

Parameter	Setting
Data normalization	Min–Max Scaling
Look-back window	7 observations
Wavelet family	Daubechies (db4)
Wavelet decomposition level	3
Wavelet coefficients	Approximation and Detail coefficients
LSTM Layer 1	128 units (return_sequences = True)
LSTM Layer 2	64 units (return_sequences = False)
Output layer	Dense (1 neuron)
Optimizer	Adam
Learning rate	0.001 (default Adam learning rate)
Loss function	Mean Squared Error (MSE)
Batch size	32
Training epochs	100
Prediction task	One-step-ahead forecasting

**Table 2:** Comparative Analysis of Forecasting Errors.

Model	Root Mean Square Error (RMSE)
Multilayer Perceptron (MLP)	0.008632
Long Short-Term Memory (LSTM)	0.008232
Convolutional Neural Network (CNN)	0.008297
Wavelet-MLP	0.008358
Wavelet-LSTM	0.008210
Wavelet-CNN	0.008223

## Data Availability

Data available upon request.

## References

[1] Harvey C.R. (1995). Predictable risk and returns in emerging markets. Rev. Financ. Stud..

[2] Frimpong J.M., Agyemang O.S. (2006). Modelling and Forecasting Volatility of Returns on the Ghana Stock Exchange Using GARCH Models. Am. J. Appl. Sci..

[3] Antwi S., Mills E.F.E.A., Mills G.A., Zhao X. (2012). The impact of dividend announcement on share price behavior in Ghana. Int. J. Bus. Soc. Sci..

[4] Engle R.F. (1982). Autoregressive conditional heteroskedasticity with estimates of the variance of United Kingdom inflation. Econom. J. Econom. Soc..

[5] Chen J.F., Chen W.L., Huang C.P., Huang S.H., Chen A.P. (2016). Financial time-series data analysis using deep convolutional neural networks. Proceedings of the 2016 7th International Conference on Cloud Computing and Big Data (CCBD).

[6] Tsang G., Deng J., Xie X. (2018). Recurrent neural networks for financial time-series modelling. Proceedings of the 2018 24th International Conference on Pattern Recognition (ICPR).

[7] Tang Q., Shi R., Fan T., Ma Y., Huang J. (2021). Prediction of financial time series based on LSTM using wavelet transform and singular spectrum analysis. Math. Probl. Eng..

[8] Tweneboah O.K., Ohene-Obeng K.A., Mariani M.C. (2025). Characterization and Prediction of the Ghana Stock Exchange Composite Index Utilizing Bayesian Stochastic Volatility Models. Risks.

[9] Panna S. (2018). An empirical investigation of the relationship between stock market returns and volatility in India. Theor. Appl. Econ..

[10] Brownlee J. (2016). Time Series Forecasting as Supervised Learning. Machine Learning Mastery. https://machinelearningmastery.com/time-series-forecasting-supervised-learning.

[11] Dorffner G. (1996). Neural networks for time series processing. Neural Netw. World.

[12] Yang J., Nguyen M.N., San P.P., Li X., Krishnaswamy S. Deep convolutional neural networks on multichannel time series for human activity recognition. Proceedings of the International Joint Conference on Artificial Intelligence.

[13] Graps A. (1995). An introduction to wavelets. IEEE Comput. Sci. Eng..

[14] Zhang D., Zhang D. (2019). Wavelet transform. Fundamentals of Image Data Mining: Analysis, Features, Classification and Retrieval.

[15] Pathak R.S. (2009). The Wavelet Transform.

